# The Malaysian Workplace Bullying Index (MWBI): A new measure of workplace bullying in Eastern countries

**DOI:** 10.1371/journal.pone.0223235

**Published:** 2020-01-23

**Authors:** Sharon Sam Mee Kwan, Michelle R. Tuckey, Maureen F. Dollard

**Affiliations:** 1 Centre for Workplace Excellence, School of Psychology, Social Work and Social Policy, University of South Australia, Adelaide, South Australia, Australia; 2 Faculty of Psychology and Education, Universiti Malaysia Sabah, Kota Kinabalu, Sabah, Malaysia; University of Florence, ITALY

## Abstract

Workplace bullying is a significant cause of stress at work. Existing studies, primarily based on Western-oriented frameworks and instruments, have largely overlooked the role of culture. This oversight questions whether understandings generated from those studies can be generalised to employees working in Eastern countries, which differ on important cultural dimensions. To date, there is no Eastern-based instrument for measuring workplace bullying. In two studies, we developed and validated such a measure: the Malaysian Workplace Bullying Index (MWBI). Study 1 entailed a content validation of bullying behaviours via written records (diaries) completed by Malaysian bullying victims. The 19 validated behaviours formed the basis of Study 2, with additions from the wider literature. Study 2 used survey data collected at three time-points from Malaysian employees exposed to bullying at work. The final result was an 18-item scale with two nine-item factors: work-related bullying and person-related bullying. Overall, the MWBI is a psychometrically sound measure of workplace bullying in Eastern workplaces.

## Introduction

Workplace bullying is a major work stressor. A recent meta-analysis revealed a number of negative job-related and health and well-being consequences of exposure, such as a range of mental and physical health problems, elevated burnout, increased intention to leave, reduced job satisfaction, and diminished organisational commitment [1]. Given the severe impacts, many studies have been conducted to investigate issues relating to workplace bullying. Nearly 95% of the research so far is from Western countries, consisting mostly of Caucasian samples [2], and the most widely-used measurement tools [2] have evolved from theories, models, and research from Western cultural perspectives. It is unwise to assume, however, that the dominant understanding and ways of measuring workplace bullying are equally applicable to employees and organisations in Eastern countries. For example, the understanding of ‘I love my job’ differs amongst employees from different countries [3, 4]. While this statement would be common among English native speakers, in Spain [4] and Malaysia [5] the word ‘love’ refers to people.

In terms of workplace bullying specifically, cultural differences can alter the experience, perception, and meaning of this phenomenon. Eastern-based research has revealed different notions of bullying. Malaysian employees view bullying, particularly work-related bullying behaviors, as common and expected–a form of institutionalised mistreatment based on the power hierarchy in Malay culture, and physical intimidation is not a factor [6]. Likewise, Loh, Restubog, and Zagenczyk [7] argued that Eastern employees perceive workplace bullying as a common practice that forms part of the organisational culture. According to Jacobson, Hood, and Buren [8] extreme assertiveness across high power distance leads to higher prevalence of bullying, based on the power inequality between the perpetrator and target. In Japan, bullying consists of indirect behaviors such as teasing, ignoring and verbal threats [9] which function as a kind of social control [10]. In contrast, most Western research studies have concluded that bullying is unacceptable and a chronic organisational stressor [11, 12], rather than accepting it as a form of inherent social and organisational control. Although national culture plays a crucial role in workplace bullying [13, 14], this influence remains largely overlooked in measuring employees’ perceptions of bullying. Although a number of studies have been conducted in Malaysia more than a decade ago [15, 16, 17, 18, 19], these studies used Western psychological concepts, measures, and models to explain workplace bullying. This oversight raises the need for a new measurement tool to more accurately assess the construct of workplace bullying in Eastern countries. To date there is no such instrument. To meet this need, the aim of this study was to develop and validate a bullying tool which could be applied within multi-cultural Eastern society–the Malaysian Workplace Bullying Index (MWBI).

In addition, previous bullying investigations have focused on the individual level [2]. The individual has been the main unit of analysis [20] with a focus on the perceptions of the targets [21]. Little is known about the consensus between team members (groups) about organisations or group level factors that predict workplace bullying. Thus, previous studies which were primarily conducted at the individual level might be insufficient to assess organisational‐ and team‐level predictors of workplace victimisation [22]. Further, the lack of multilevel investigations of bullying has limited notions to assess organisational climate at organisational level [23]. Hence, there is a crucial need to focus on multilevel analysis in line with calls for group-level assessments of workplace bullying [24]. To date, no measurement has been developed to take the organisational level into account. We addressed this gap by validating the new MWBI measure at both the individual and group (organisation) levels. This multilevel approach has not been considered in relation to bullying and is relatively new in psychometric testing more broadly and is therefore valuable for knowledge advancement. Thus, our objective was to build an integrated workplace bullying measurement tool from a multilevel standpoint.

## Workplace bullying in Eastern countries

Although there are variations amongst definitions of bullying, the key characteristics include behavior that is carried out repeatedly over time, sustained by a power imbalance, and causes harm to the target [25]. Although the conceptualisation of bullying originated in Western countries (e.g., Leymann’s seminal work) [26], workplace bullying is a global issue, arising in Eastern countries partly as a function of high power distances [27, 28, 29] and individualism-collectivism dimensions [7, 8, 30].

During the 1980s, a few Eastern scholars began to study the concept of workplace bullying. The word *ijime* was used in Japan which refers to bullying and entails harassment of colleagues [9]. Japanese society places high expectations on individuals, and employees often suffer low self-satisfaction; consequently, bullying has also been used as a means to drive employees out of an organisation [31]. In Korea, the term *tae-wum* (or *tae-wu-gi)* which means ‘to burn something’ has featured in the media [28]. This term is used for harmful behavior amongst nursing staff and consisted of physical and psychological abuse of nurses by superior nurses or doctors. It involved physical violence, social isolation, verbal abuse and unbearable work tasks [29], and it has been justified by medical superiors as a way to increase staff efficiency [28].

A handful of similar investigations have been conducted in China [32, 33] and other countries with Chinese-based cultures such as Taiwan [34, 35] and Hong Kong [36]. Chinese culture is characterised as having high power distance [27] which is reflected in hierarchical relationships between higher managements and subordinates [37]. Therefore, bullying is seen unavoidable for employees in Chinese-based countries because most perpetrators are superiors. For example, Chen [36] found that in Hong Kong, superiors were most often responsible for bullying which commonly involved verbal abuse, appointing victims to tasks no one wants, and ignoring victims; not surprisingly most victimised employees suffer in silence.

Research studies in both India and Pakistan reported that in the medical industry, perpetrators are usually from higher positions such as senior doctors [38] or consultants [39], and this power imbalance decreases the likelihood that victims will seek assistance [38, 39]. In India, high power distance and hierarchy are factors which contribute to the complexity in Indian society and workplaces [40]. These two elements arguably influence the occurrence of workplace bullying. Although organisations may have policies with provisions for bullying complaints, complainants are perceived as trouble-makers [40]. Little to no effective action is taken by the organisations to help victims and witnesses. This underscores the importance of building an organisational environment wherein employees are clear and aware about acceptable and unacceptable behaviours (e.g. workplace bullying), about options available to deal with such behaviours [40], and in which meaningful action is taken to stop such behavior [41].

In the Philippines, bullying is also not commonly addressed because complaining is considered to be an admission of weakness, making the victim even more vulnerable [42]. Likewise, Loh et al. [7] found that Singaporean employees, working a high power-distance society, are less likely to respond proactively to bullying compared to Australian employees.

Workplace bullying is an emerging concept in Malaysia too. Bullying is reported by Kwan et al. [6] as part of the dominant culture of Malaysian organisations. Employees feel pressure to acquiesce to bullying situations because such behavior represents power and authority within the organisation. Indeed, based on work by International Business Machines (IBM), Malaysia recorded the highest difference in power distance among 76 countries and regions [43]. Although the Malaysia Department of Safety and Health has provided a guideline for prevention violence at work, there is a shortage of information about the duration, situations and behaviors that constitute bullying in Malaysian organisations.

Besides power distance, the individualism versus collectivism dimension of culture is theorised to influence workplace bullying. A study has been conducted to examine the impact of culture on the acceptability of workplace bullying in six continents: Anglo, Confucian Asia, Eastern Europe, Latin America, Sub-Saharan Africa and South Asia [44]. The results showed that workers from Confucian Asian societies–collectivist cultures–are more likely to tolerate bullying compared to workers from other (individualist) continents. The relatively high level of performance orientation which values working together as members of an organisation (institutional collectivism), or in-group (collectivism), in Confucian Asia societies renders employees to endure unpleasant work practices and tolerate bullying [44]. Therefore, when societies have high in-group collectivism, bullying is predicted to be low because a collectivistic culture tends to show a higher level of care and concern for others. However, when in-group collectivism interacts with power distance, collectivist cultures that have high power distance might use group-type bullying (i.e. mobbing) because the power of the group supersedes an employee’s individualistic goals [8]. In contrast, workers in cultures with high levels of individualism and low power distance will be more likely to engage in resistance-based responses toward the perpetrator of the bullying [45]. Workers in individualistic cultures focus on the role of individual choice, personal freedom and self-actualisation [46], thus, they will be more likely to become attentive to, and reflect on, such bullying behaviors as negative and act upon to deal with these behaviors [45].

Most Eastern countries have a collectivist culture [43, 47] which means that bullying becomes a group issue, not just an issue for the individual [48]. In Korea, bullying is a group act which referred to ‘wang-tta’ [49]. Group benefits and strong social bonds play a very important role in Korean society. Any individual (e.g. worker) who appears to be different or to be a threat to the group can easily be targeted. These discriminative acts are likely to appear from the group, which may take the form of group conflict escalating into bullying [49].

In summary, acts of bullying may be viewed differently in different cultural contexts [8, 49]. While Western scholars define bullying as an unresolved conflict in the organisation [50], bullying in Malaysia is considered to be a social mechanism to maintain existing power differences between people in higher positions and subordinate employees [6]. In other Eastern countries, workplace bullying is a group issue [49] and bullying has been described as a way to maintain hierarchy and order [27, 32, 38, 39, 51]. Because of these different culturally-based perspectives in the conceptualisation of bullying, Western-origin measurement instruments may not necessarily be generalised to Eastern worksites.

## The present study

In the development of a new method for measuring workplace bullying, we sought to adopt a mixed-methods approach [52] in two different research studies. Study 1 is a qualitative study using diary writing providing content validation of bullying behaviors identified in Kwan et al.’s [6] interviews with Malaysian workers. The 19 behaviors derived from the interviews were further validated through diary writing. Diary studies allow researchers to gather data in people’s life contexts and thus serve as a useful way of recording events, thoughts, feelings and behaviors in their own words [53]. The behaviors that being validated from the diary writing were used to form an index that was further validated in Study 2 –a quantitative study involving a three-wave survey of employees nested within 50 teams/organisations. Based on the results, the Malaysia Workplace Bullying Index (MWBI), consisting of 18 items, was developed as a reliable and valid tool to investigate workplace bullying in Eastern cultures.

## Study 1

The research study has received ethics approval from the University of South Australia’s Human Research Ethics Committee. In Study 1, member checking [54] was used to address content validity of the 19 bullying behaviors identified by Kwan et al. [6] from interviews with Malaysian workers. Although participants were asked to record bullying experiences in a diary against the inventory of behavioral items, they were also encouraged to record new bullying behaviors.

### Methods

#### Participants

Participants (n = 8) included targets and witnesses of recent workplace bullying who were willing to participate in diary writing about bullying events. Written consent was obtained from all participants prior to them recording their current experiences in diaries. The sample was comprised of current targets and witnesses (n = 6, 30%) and current witnesses only (n = 2, 10%). There were four females and four males, aged from 23 to 37 (*M* = 31.0, *SD* = 5.1). Half of the participants were employees from government sector and half in the private sector as follows: administrative support staff (n = 4, 50%), and one each (12.5%) were academic staff, executive, non-executive, and teacher.

#### Diary writing

A diary template was provided to participants in a sealed envelope, containing the definition of workplace bullying and list of 19 bullying behaviors identified by Kwan et al. [6]. The definition is “Workplace bullying is intimidating, persecuting, or offending behavior with the intention to harm and victimize someone due to a power imbalance; this behavior causes physical and psychological distress to the target of the bullying” (2014, p. 192 [6]). Participants were requested to record episodes of bullied at work (or incidents they witnessed) from on a daily basis if any from the list provided and the accounts being described according to the template. Details included date and time, nature of the bullying behaviors, organisational status of the perpetrator, and coping strategies used. The participants were asked to include additional bullying behavior not included in the list, and record responses for those behaviors in the same way. The diaries were collected weekly, whereupon a new diary template was provided. The diary writing continued for one month–that is, four weekly templates were completed.

#### Analysis

The data analysis was started after the collection of diary templates from all the participants. Firstly, participants who encountered/ witnessed bullying behaviors were identified. Secondly, the authors identified the nature of the bullying behaviors by calculating the total of episodes of the behaviors based on the occurrence dates reported by the participants in the diary. Thirdly, the position of the bullying perpetrators could also be identified by summing the number of organisational status (employer, manager, supervisor, senior, co-worker or other) that selected by the participants. Finally, the authors recorded the frequency of the bullying behaviors reported by the participants in the diary template. For instance, a few participants reported bullying episode almost every day and every week and some participants encountered bullying exposure occasionally such as during meetings at work during the four-week diary writing period. All the results are reported in a table.

### Results

A total of 47 bullying episodes were recorded by the participants. Of the eight participants, four reported that they faced bullying occasionally, but the other four experienced frequent (weekly or daily) exposure (refer to Table 1). The bullying incidents were evenly spread among the participants. Four participants encountered five incidents, one participant encountered six incidents and three participants encountered seven bullying incidents. All participants reported that they have been requested to do work which is supposed to be done by other co-workers Table 1 shows the number of episodes of bullying, positions of perpetrators, and frequencies of exposure according to the targets and witnesses from Study 1. As shown, each behavior was experienced at least once during the 47 bullying episodes recorded by participants. None of the participants reported new bullying behaviors beyond the list provided, most likely because the key issues had already been captured in the interviews. The behavior ‘Being requested to do work which is supposed to be done by other co-workers’ was reported most frequently (10 episodes, 21.3%), followed by ‘Being requested to do unnecessary work which is not relevant to the job description’; and ‘Being scolded without relevant reason’ with five episodes reported (10.6%). The target and witness participants also reported that all perpetrators held a higher position (100%) in the formal organisational hierarchy than the target.

**Table 1:** Number of bullying episodes, positions of perpetrators and frequencies of bullying behaviours according to current targets and witnesses.

	**Bullying behaviours**	**No. of episodes**
1.	Being requested to do work which is out of the job scope	1
2.	Being requested to do work that is not within one's ability	1
3.	Being requested to do unnecessary work which is not relevant to the job description	5
4.	Being requested to do an excessive amount of work	1
5.	Being requested to work overtime without pay	1
6.	Being requested to do work which is supposed to be done by other co-workers	10
7.	Being asked to do work alone without assistance	1
8.	Being instructed to do work without guidance	2
9.	Being forced to do work	3
10.	Being forced to do work to meet deadlines	2
11.	Having credit for the work taken by someone else	2
12.	Being coerced or threaten to do work	1
13.	Being threatened that privileges will be taken away by someone else	1
14.	Being wrongly blamed if something is wrong	3
15.	Being taken advantage of	1
16.	Being scolded without relevant reason	5
17.	Being make fun of	1
18.	Being talked about behind one’s back	3
19.	Having rumours spread about oneself	3
	**Total**	**47**
	**Position of perpetrator**	**No. of participants**
	Employer	5
	Manager	3
	Supervisor	3
	Senior	5
	Total	16
	**Frequency of bullying behaviours**	**No. of participants**
	Occasionally	4
	Weekly	2
	Daily	2
	Total	8

### Discussion

The aims of Study 1 were to establish support for the content validity of 19 bullying behaviors identified by Kwan et al. [6], before proceeding to a larger-scale quantitative validation study in Study 2. The results indicated that all 19 behaviors are experienced by targets, and together they represent comprehensive coverage of the nature of bullying. All exposures to bullying during the four-week diary writing period were from those more senior in the hierarchy–none were from employees at the same level or below in the hierarchy. This result supports the proposition that power distance plays a role in the occurrence of workplace bullying. In sum, all 19 bullying behaviors were validated through member-checking by targets and witnesses; these behaviors formed the basic psychometric items of bullying subsequently used in Study 2.

## Study 2

The nature of Study 2 tended to further the index validation process. The validation strategies focused on construct, convergent, concurrent and predictive validity of the 19-item measure–the Malaysian Workplace Bullying Index (MWBI). First, to establish construct validity, we employed factor analysis and examined internal consistency [55]. Second, convergent validity is demonstrated when an instrument correlates well with a measurement (Negative Acts Questionnaire- Revised) that has previously been validated and measured for the same construct. Third, concurrent validity is demonstrated when the new measurement could predict other outcomes using the data that are collected at the same time. In the current study, scores on the MWBI were correlated with an established measure of workplace bullying, as well as two health outcomes (emotional exhaustion and posttraumatic stress), all expected to be positively associated with the MWBI [56, 57]. Forth, predictive validity is demonstrated by examining the relationship between MWBI with the health outcomes overtime.

### Methods

#### Participants

The first author met with representatives from 50 different organisations across diverse industries in Sabah, Malaysia to discuss participation in the project. Following approval, one department or team from each organisation, consisting of at least 10 employees, was invited to participate. Written informed consent was obtained from all organisations and participants in Study 2. Questionnaires were distributed at three time-points to 500 workers (10 per team) nested within 50 teams, secured in separate envelopes to maintain confidentiality. The time lag between waves was four months, considering that bullying behaviors occur over a period of time [58]. At each time-point, after one week the questionnaires were collected by for analysis. At Time 1, 500 employees responded (100% response rate); 461 of the same participants responded at Time 2 (92.2% response rate); and at Time 3 the figure was 438 (87.6% response rate). Only data from participants who had some exposure to bullying behaviors were analysed, giving revised totals of: Time 1 = 444, Time 2 = 414, and Time 3 = 392. Table 2 shows the demographic characteristics for participants at each of the three-time points.

**Table 2:** Main characteristics of the participants for three-time points.

Demographic Variables	Time 1		Time 2		Time 3	
	n = 444	%	n = 414	%	n = 392	%
**Gender**						
Male	159	35.8	147	35.5	139	35.5
Female	285	64.2	267	64.5	253	64.5
**Age (ranged 16–60)**						
Mean	38		32		32	
Standard Deviation	8.2		8.1		8.1	
**Marital Status**						
Single	214	48.2	202	48.8	190	48.5
Married	222	50.0	204	49.3	194	49.5
Divorced	7	1.6	7	1.7	7	1.8
None (missing data)	1	0.2	1	0.2	1	0.2
**Type of company**						
Government	232	52.3	216	52.2	206	52.6
Semi government	20	4.5	18	4.4	16	4.1
Local-owned private company	129	29.0	121	29.2	115	29.3
Foreigner-owned private company	34	7.7	31	7.5	28	7.1
Others	29	6.5	28	6.7	27	6.9
**Employment positions**						
Employer	13	2.9	12	2.9	11	2.8
Manager	16	3.6	15	3.6	13	3.3
Supervisor	14	3.2	14	3.4	14	3.6
Officer	72	16.2	69	16.7	65	16.6
None-executive	33	7.4	29	7.0	26	6.6
Executive	30	6.8	29	7.0	27	6.9
Specialist	12	2.7	12	2.9	11	2.8
Support staff	188	42.3	177	42.8	169	43.1
Others	55	12.4	47	11.3	47	12.0
None (Missing data)	11	2.5	10	2.4	9	2.3
**Participation in Union**						
Members	129	29.1	123	29.7	121	30.9
Non-member	307	69.1	283	68.1	264	67.3
None (missing data)	8	1.8	8	1.9	7	1.8

#### Questionnaire

Workplace bullying. As recommended by Neall and Tuckey [2] and Nielsen and Einarsen [1], we used a combination of the definition and behavioral exposure methods to assess workplace bullying in the MWBI. First, a definition of bullying based on Kwan, Tuckey and Dollard [6] was presented. Second, the 19-item bullying behaviors survey (e.g., “Being talked about behind one’s back”) validated in Study 1 was administered, rated on a 5-point Likert-type scale: (0) *never*, (1) *now and then*, (2) *monthly*, (3) *weekly*, and (4) *every day*. To assess convergent validity, the Negative Acts Questionnaire-Revised (NAQ-R) [59] was also included in the questionnaire. It is comprised of 22-items (e.g., “Having your opinions ignored”), rated on a scale from (1) *almost never* to (5) *daily*.

Emotional exhaustion. The Maslach Burnout Inventory (MBI) [60] was used to assess emotional exhaustion using five items, based on a 7-point Likert scale. A sample item is “I feel used up at the end of the work day”. The response scale ranged from (1) *never* to (7) *always*.

Posttraumatic stress. The Impact of Event Scale-Revised (IES-R) [61] which consists of 22 items, was used to assess symptoms of posttraumatic stress. The three major symptom clusters of post-traumatic stress are assessed: intrusion (e.g., “Any reminder which brought back feelings about it”), avoidance (e.g., “I avoided letting myself become upset when I thought about it or was reminded of it”), and hyper-arousal (e.g., “I felt irritable and angry”). Items were rated on a 5-point Likert-type scale, from (0) *not at all* to (5) *extremely*.

#### Analysis

Given the nested nature of the data, reflecting the hierarchical structure of most organisations, multilevel exploratory factor analysis (MEFA) and multilevel confirmatory factor analysis (MCFA) were conducted using M*plus* Sofware Version 6.0 [62]. MEFA explored the dimensions or factors of the 19 bullying behaviors resulting from Study 1 at Times 1 and 2. The bullying items resulting from MEFA were then confirmed through MCFA at Time 3.

The extraction method used was Kaiser’s criteria (eigenvalue > 1 rule; see [63]) and the cumulative percent of variance was assessed, using a threshold of 50% [64]. In addition, five fit indices were considered: (1) a non-significant value of chi-square (χ^2^); (2) a small degree of freedom (df) [65]; (3) the value for comparative fit index (CFI) must be more than .90 [66, 67]; (4) the value for root mean square error of approximation (RMSEA) is equal to or less than .08 [66]; and (5) standardised root mean square residual (RMSR) must be less than .08 [68]. Finally, factor loadings were examined. Items with loadings greater than .50 [69] were included in the index as being practically significant to the factor [70].

The intra-class correlation coefficient (ICC) was used to assess group-level variation in MWBI scores [71]. The median value of ICC is .12 [72] which we adopted as the meaningful variance threshold at the group (organisation) level [65]. Furthermore, Cronbach alpha coefficients were calculated for MWBI scores to test the internal consistency of the index in both individual and group levels. The criterion for high internal consistency is above .80 [73].

To examine measurement validity, Pearson’s correlation coefficients were calculated at Time 3 between the MWBI and (a) an alternate, established measure of workplace bullying (NAQ-R) for examining convergent validity; and (b) two health outcomes (emotional exhaustion and posttraumatic stress) for examining concurrent validity. To examine predictive validity, these relationships were examined over time using the MWBI scores at Time 2 and the health outcomes scores at Time 3.

### Results

#### Construct validity

Multilevel exploratory factor analysis. A series of MEFAs with two-, three-, and four-factor solutions for both within- and between-group levels were conducted for the MWBI items at Time 1 and Time 2. Summary statistics for the Time 1 MEFA including all 19 items are shown in Table 3.

**Table 3:** Multilevel exploratory factor analysis for 19 items at Time 1.

Factors	*χ*^2^	*df*	CFI	RMSEA	RMSR (Within/ Between)
1W1B	892.50	304	0.90	0.06	0.14 0.47
2W1B	391.90	286	0.98	0.02	0.06 0.47
3W1B	291.33	269	0.99	0.01	0.04 0.47
4W1B	255.94	253	1.00	0.00	0.03 0.47
1W2B	1081.19	286	0.87	0.07	0.14 0.38
2W2B	427.67	268	0.97	0.03	0.06 0.38
3W2B	290.64	251	0.99	0.01	0.04 0.38
4W2B	245.74	235	0.99	0.01	0.03 0.38
1W3B	1086.98	269	0.87	0.08	0.14 0.38
2W3B	419.82	251	0.97	0.03	0.06 0.38
3W3B	278.90	234	0.99	0.02	0.04 0.38
4W3B	233.09	218	0.99	0.01	0.03 0.38
1W4B	1068.79	253	0.87	0.08	0.14 0.35
2W4B	405.52	235	0.97	0.04	0.06 0.35
3W4B	264.50	218	0.99	0.02	0.04 0.35
4W4B	218.80	202	0.99	0.01	0.03 0.35

Note: χ^2^ = Chi-Square; *df* = degree of freedom; CFI = comparative fit index; RMSEA = root mean square error of approximation; RMSR = standardised root mean square residual

Based on the fit statistics, the three within and one between (3W1B) factor model most closely represented the data: χ^2^ = 291.33, *df* = 269, CFI = .99, RMSEA = .001 and RMSR (W/B) = .04/.47, although a number of other models had excellent fit statistics. Information regarding the eigenvalues and accumulated percentage of explained variance (%) at Time 1 is presented in Table 4. The eigenvalue for three within (3W) group variance is 1.17 (more than one) [74] and the accumulated percentage is 69.72% which must be at least 50% [64]. The eigenvalue for one between (1B) group variance is 10.39 and the accumulated percentage of explained variance is 54.71%. However, five items (items 9, 11, 12, 15 and 18) had cross loadings exceeding the cut-off value of .50 [69]. Although some researchers suggested dropping items with cross loadings and rerunning the analysis, too many deleted items may influence the integrity of the data [75]. Accordingly, we checked the loadings for the models with two within and one between (2W1B) group factors (see Table 5). Inspecting the loadings, only item 9 “Being forced to do work” had a cross-loading exceeding the threshold. The fit indices for the two within one between (2B1W) factor model showed good fit: χ^2^ = 391.90, df = 286, CFI = .98, RMSEA = .02 and RMSR (W/B) = .006/.47 (see Table 3). Likewise, the eigenvalue for two within (2W) components is 2.72 and the accumulative percentage of variance is 63.55% (see Table 4). Based on these results, item 9 was deleted (as suggested by [76]) and multilevel EFA was conducted on the remaining 18 items.

**Table 4:** Eigenvalues, percentage of explained variance (%) and accumulated percentage of explained variance for within and between sample correction matrix for 19 items at Time 1.

Time	Level	Components	Eigenvalues	Percentage of explained variance (%)	Accumulated percentage of explained variance (%)
1	Within	1	9.35	49.22	49.22
		2	2.72	14.33	63.55
		3	1.17	6.17	69.72
		4	0.90	4.78	74.50
	Between	1	10.39	54.71	54.71
		2	1.22	6.42	61.13
		3	1.00	5.28	66.41
		4	0.71	3.78	70.19

**Table 5:** Overall model fit for multilevel exploratory factor analysis for 18 items at Time 1 and Time 2.

Factors	Time	*χ*^2^	*df*	CFI	RMSEA	RMSR (Within/ Between)
1W1B	1 2	855.85 2468.63	270 270	0.89 0.28	0.07 0.14	0.14/0.46 0.27/0.25
2W1B*	1 2	344.15 407.68	253 253	0.98 0.95	0.03 0.04	0.06/0.46 0.05/0.25
3W1B	1 2	263.11 287.81	237 237	0.99 0.98	0.01 0.02	0.04/0.46 0.04/0.25
4W1B	1	226.59	222	0.99	0.01	0.03/0.46
	2	No convergence				
1W2B	1 2	1016.00 2476.27	253 235	0.86 0.28	0.08 0.14	0.14/0.37 0.27/0.16
2W2B	1 2	366.68 392.80	236 236	0.97 0.94	0.03 0.04	0.06/0.37 0.05/0.16
3W2B	1 2	261.09 267.93	220 220	0.99 0.98	0.02 0.02	0.04/0.37 0.04/0.16
4W2B	1 2	215.04 No Convergence	205	0.98	0.01	0.03/0.37
1W3B	1 2	1018.37 No Convergence	237	0.86	0.08	0.14/0.37
2W3B	1 2	357.58 No convergence	220	0.97	0.03	0.06/0.37
3W3B	1 2	249.49 No convergence	204	0.99	0.02	0.04/0.37
4W3B	1 2	202.51 No convergence	189	0.99	0.01	0.03/0.37
1W4B	1 2	992.78 No convergence	222	0.86	0.08	0.14/0.36
2W4B	1 2	341.84 No convergence	205	0.97	0.03	0.06/0.36
3W4B	1 2	234.86 No convergence	189	0.99	0.02	0.04/0.36
4W4B	1 2	188.30 No convergence	174	0.99	0.01	0.03/0.36

Note: χ^2^ = Chi-Square; df = degree of freedom; CFI = comparative fit index; RMSEA = root mean square error of approximation; RMSR = standardised root mean square residual

*The 2 Within (W) - 1 Between (B) factor model was deemed the best fitting model.

The MEFA for the remaining 18 items was run on data from Time 1 and Time 2, as shown in Table 5. Model 2, which contained two within-group factors and one between-group factor (2W1B), fit the data very well: Time 1, χ^2^ = 334.15 *df* = 253, CFI = .98, RMSEA = .03 and RMSR (W/B) = .06/.46; Time 2, χ^2^ = 407.68, *df* = 253, CFI = .95, RMSEA = .04 and RMSR (W/B) = .05/.25. Looking to the more stringent cut-off criterion for eigenvalues and accumulated percentage of explained variance, as shown in Table 6, Model 2 with two within-group factors and one between-group factor (2W1B) was the best model. For the within level, the second component has an eigenvalue more than one (2.67, T1: 5.02, T2) and above 50% accumulated percentage of explained variance (63.48%, T1; 59.38%, T2). For the between level, the first component has high eigenvalues of 10.09 at Time 1 and 10.84 at Time 2, and accumulated percentage of explained variance values of 56.09% and 60.25% for Times 1 and 2 respectively.

**Table 6:** Eigenvalues, percentage of explained variance (%) and accumulated percentage of explained variance for within and between sample correction matrix at for 18 items at Time 1 and Time 2.

Time	Level	Components	Eigenvalues	Percentage of explained variance (%)	Accumulated percentage of explained variance (%)
1	Within	1	8.75	48.63	48.63
		2	2.67	14.85	63.48
		3	1.06	5.93	69.41
		4	0.90	5.01	74.42
	Between	1	10.09	56.09	56.09
		2	1.11	6.20	62.29
		3	0.97	5.43	67.72
		4	0.70	3.92	71.64
**2**	Within	1	5.66	31.49	31.49
		2	5.02	27.89	59.38
		3	0.95	5.30	64.68
		4	0.86	4.81	69.49
	Between	1	10.84	60.25	60.25
		2	1.55	8.66	68.91
		3	0.68	3.82	72.73
		4	0.65	3.62	76.36

Therefore, the model with two within-group and one between-group factor (2W1B) was deemed the best fitting model. Based on within-group variance, bullying behaviors could be distinguished into two factors. The first factor comprised nine items, from item 1 to item 9, all off which describe work-related bullying behaviors. The second factor comprised another nine items, from item 10 to item 18, reflecting person-related bullying. Table 7 illustrates the 18 items with their respective factors and factor-loadings. The table shows that all the standardised factor loadings were in the estimated range from .50 to .97, exceeding the cut-off value of .50 [69]. Based on the between-group variance, bullying behaviors manifest as one overall factor.

**Table 7:** Factors loadings for 2Within and 1Between (2W1B) Model for 18 items at Time 1 and Time 2.

	Item	Time 1		Time 2	
		Factor 1	Factor 2	Factor 1	Factor 2
	**Work-related bullying**				
1.	Being requested to do work which is out of the job scope	**0.69**	0.45	**0.63**	0.39
2.	Being requested to do work that is not within one's ability	**0.76**	0.38	**0.92**	0.23
3.	Being requested to do unnecessary work which is not relevant to the job description	**0.82**	0.36	**0.83**	0.19
4.	Being requested to do an excessive amount of work	**0.78**	0.41	**0.80**	0.24
5.	Being requested to work overtime without pay	**0.71**	0.47	**0.92**	0.33
6.	Being requested to do work which is supposed to be done by other co-workers	**0.74**	0.43	**0.64**	0.30
7.	Being asked to do work alone without assistance	**0.91**	0.47	**0.84**	0.12
8.	Being instructed to do work without guidance	**0.84**	0.41	**0.76**	0.22
9.	Being forced to do work to meet deadlines	**0.81**	0.40	**0.50**	0.20
	**Person-nature bullying**				
10.	Having credit for the work taken by someone else	0.46	**0.70**	0.37	**0.55**
11.	Being coerced or threaten to do work	0.45	**0.79**	0.13	**0.67**
12.	Being threatened that privileges will be taken away by someone else	0.39	**0.81**	0.17	**0.66**
13.	Being wrongly blamed if something is wrong	0.38	**0.76**	0.23	**0.66**
14.	Being taken advantage of	0.33	**0.78**	0.22	**0.80**
15.	Being scolded without relevant reason	0.42	**0.74**	0.35	**0.97**
16.	Being make fun of	0.45	**0.78**	0.23	**0.67**
17.	Being talked about behind one’s back	0.35	**0.78**	0.17	**0.66**
18.	Having rumours spread about oneself	0.44	**0.77**	0.33	**0.67**

In addition, the intra-class correlation coefficient (ICC) was used to determine potential group influences [71] which show the between group level effect when working with multilevel data [65]. According to Bliese [77], a small ICC value is less than .10 and a large ICC value is more than .70. At Time 1, the ICC value for work-related bullying is .07 and the person-nature bullying is .05, indicating 7% of the variance in work-related bullying and 5% of the variance in person-nature bullying was due to organisation level. At Time 2, the ICC values increased to.12 and .16 for work-related bullying and person-nature bullying, indicating 12% and 16% respectively, surpassing the threshold of .12 [72].

Multilevel Confirmatory Factor Analysis. A multilevel confirmatory factor analysis (MCFA) was conducted using the Time 3 data to confirm the factor structure of the 18-item MWBI. The model with two within-groups factors and one between-groups factor models had satisfactory fit to the data: chi square (χ^2^) = 724.05; degrees of freedom (df) = 269; CFI = .91; and RMSEA = .06. Also, the standardised root mean square residual (RMSR) was .06 at the within level and .08 at the between level (.08).

Reliability. Cronbach alpha coefficients were calculated for the MWBI at Time 3. At the individual level, alpha was .95. For the two factors–work-related bullying and person-related bullying–the values were .93 and .94 respectively. At the group level, the alpha coefficient was .89; with values of .92 and .91 for work-related bullying and person-related bullying respectively. Overall, these values indicate excellent internal consistency.

#### Convergent and concurrent validity

In relation to the convergent validity of the MWBI, as expected the correlation at Time 3 with the alternate bullying measure (NAQ-R) was *r* = .72, *p* < .01. For the health outcomes in relation to concurrent validity, the correlations at Time 3 were also in line with predictions: *r* = .50, *p* < .01 for emotional exhaustion and *r* = .70, *p* < .01 for posttraumatic stress. Similarly, high correlations (ranging from .56 to .84) were observed between the MWBI sub-scales and both health outcomes.

#### Predictive validity

In order to establish predictive validity, we calculated the correlations between the MBWI at Time 2 and the health outcomes at Time 3. The correlation for emotional exhaustion was *r* = .60, *p* < .01, and for posttraumatic stress *r* = .72, *p* < .01. Further, there were high correlations between the MWBI sub-scales and the health outcomes (ranging from .56 to .85).

### Discussion

The central objective of Study 2 was to establish the validity of the MWBI via longitudinal survey research at three-time points. Based on MEFA on the Time 1 and 2 data, all but one of the original 19 items were retained in the index, with two sub-factors identified at the within-group level and one at the between-group level. The factor structure was confirmed using MCFA at Time 3, and internal consistency was high in further support of construct validity. Overall, the two within- and one between- (2W1B) model had the best fit based on all criteria. In other words, at the individual level (within-group variance), bullying behaviors can be distinguished into two categories: work-related and person-related bullying. At the group level (between-group variance), however, bullying behaviors manifest as one overall category only. Study 2 established support for the convergent validity of the MWBI through a significant positive correlation with a widely-used measure of workplace bullying, indicating that the MWBI is indeed tapping the bullying construct. Study 2 also reported a strong support for concurrent and predictive validity because MWBI showed significant negative correlations with two indicators of psychological health problems (emotional exhaustion and posttraumatic stress symptoms), indicating that the MWBI can predict negative health outcomes due to bullying (at the same time and overtime).

## General discussion

Our study addressed the cultural element by examining the conceptualisation of bullying behaviors from the perspective of the culture of Malaysian employees. Einarsen et al. [25] described bullying as a multi-causal social phenomenon that includes cultural and socioeconomic factors. A study by Lutgen-Sandvik et al. [14] reported that the features of Hofstede’s cultural framework [43] which is power distance, play vital roles in the prevalence of bullying. This current study has validated bullying behaviors and identified psychometric properties of bullying using multilevel analysis within Eastern cultures. Since both Western and Eastern countries have different cultural orientations according to Hofstede [43], it is crucial to understand bullying behaviors from the standpoint of employees from Eastern organisations. This is important because any future research must consider how culture influences employees’ perceptions of bullying.

Taken together, the findings from Study 1 and Study 2 indicate that the MWBI has good psychometric properties in terms of construct, convergent, concurrent and predictive validity. The MWBI can be explained by two dimensions: 1) work-related bullying and (2) person-related bullying at the individual level. Although the finding differs with other workplace bullying measurements such as the NAQ-R (which reported physical intimation as a bullying factor), the current results of validity and reliability testing showed that the MWBI is a valid measurement tool for measuring workplace bullying. This result implied that the bullying behaviors that being understood by the Western employees based on Western measurement (NAQ-R) may not be understood and agreed by the Eastern employees particularly in the Malaysian context. The reason is culture influences perceptions on bullying [45] that may alter the meaning of bullying behaviors. As stated in DOSH [78], physical intimidation refers to physical violence and bullying refers to a form of psychological violence. Thus, it is understandable that why the Malaysian employees do not treat physical-intimating behavior as bullying. Both MWBI and NAQ-R measure an identical construct which is workplace bullying but the MWBI is more valid for measuring workplace bullying especially within the Eastern context.

In addition, the validity process of the index which consisted of content, construct, convergent, concurrent and predictive validations have avoided and resolved the issue of common method bias [79]. This current study employed a mixed-method approach which consisted of the qualitative procedure of diary-writing, and the quantitative method of a questionnaire, have yielded data from participants–and especially from the longitudinal study encompassing three-time-points. In Study 1, the diary writing reconfirmed the bullying behaviors reported by Kwan et al., [6] in addressing the content validity of the index and the data from the initial study facilitated the development of a list of items of bullying behavior for Study 2. The second study also showed accurate and reliable validation by addressing the construct validity using factor analysis of the bullying items at different time-points.

Additional evidence for concurrent and predictive validity of the MWBI was established through adverse psychological health outcomes (at the same time and overtime). Previous studies have correlated bullying with cardiovascular health [2], headaches, stomach disorders, musculoskeletal health [80], eardrums, spinal injuries [81], posttraumatic stress [11, 56], and emotional exhaustion [80, 82]. Thus, the index can be used to identify workplace bullying with other associations. In this current study, high MWBI scores were correlated with high posttraumatic stress.

Lastly, this current study has validated bullying behaviors and identified psychometric properties of bullying from the organisation level using multilevel analysis. From the current study, bullying behaviors at the group level could be manifested as one overall category. This finding means that there is a factor called bullying which can be determined at the organisational level. If a bullying exposure happens in the organisation, it is interpreted beyond the individual at an organisational level because it has organisational or group level sources. This finding has supported the notion that the shared perceptions of the work environment within the organisations might explain the bullying behaviors [21]. Work environment has influenced the occurrence of workplace bullying [41, 83]. Organisation serves as a precursor to bullying [84]. Hence, the MWBI can be applied at the organisational level to further investigate organisational mechanisms underlying bullying using multilevel analysis.

### Implications

The development and validation of the MWBI has particular implications for workplace bullying. Our overarching contribution is developing an Eastern-based index to measure workplace bullying. The research work uses qualitative data from an earlier study, along with both quantitative and qualitative data in the development of a new measure of bullying in Malaysia. It is critically important to consider bullying within the cultural context, so the study is significant in taking a first step in determining a survey for eastern cultures. Culture influences the how workplace bullying is perceived. The development and validation of the MWBI has addressed the call from Cowie et al. [85] who previously noted the absence of measurement techniques suitable for use in Eastern contexts. This current study also addressed Seo [29] who reported a lack of instruments applicable to Korean worksites. Since Malaysia and Korea share similar collectivist traditions, there was a need to develop a questionnaire that takes account of Eastern perceptions and beliefs.

Therefore, measurement of bullying by means of Western standards may not be applicable to Eastern work situations. Overall, the current study indicates that the psychometric properties of the MWBI represent a valid and useful instrument for measuring bullying, and it is applicable not only to Malaysian employees but also to employees from other Eastern countries. An important strength of the MWBI is that it assesses a wider range of different behaviors related to work-tasks. Thus, the development of the MWBI is crucial for the workplace bullying literature because a dependable psychometric measurement is a prerequisite for theoretical advancement [86].

The current study is also pertinent to the body of knowledge of workplace bullying. By using a mixed-method approach combining qualitative (diary writing) and quantitative (questionnaire) procedures, it enriched our understanding of bullying behaviors from an Eastern standpoint, and it further developed a solid questionnaire about workplace bullying. The participants who were nested in teams from different organisations added strength to the soundness of the study. Both individual and organisational levels have been taken into account in developing a checklist of items for the index, regardless of whether the bullying occurred from within or between levels. This multilevel (two level) analysis differs from a one-level analysis (individual) in which most of the previous investigations have been carried out. The multilevel approach may facilitate better explanation of the mechanisms of workplace bullying from the perspectives of whole work groups or team members in an organisation [21]. From the current study, bullying occurred in two-within and one-between factor models which means that apart from individual level, organisational level plays a role in the bullying exposure. This finding supported Kwan, Tuckey and Dollard’s work [41] which discussed the influence of organisations in limiting the escalation of bullying behaviors at work. In that sense, the current study provides new insights for future research into workplace behavior from a multilevel analysis; this can be extended to other workplace bullying research studies in Eastern and Western countries, as well as in cross-cultural research investigations–especially when the MWBI is used as the measuring tool.

## Limitations and future research

Some limitations of the current study are acknowledged. This study was constrained insofar as it surveyed only Malaysian employees. This limitation raises some uncertainty about whether the index is applicable to other populations of employees. Further investigations are required regarding the possible universal application of this index to other populations and whether it is pertinent to comparative cross-cultural studies using the MWBI and other bullying measurement tools. It is vital to test the MWBI in other collectivist countries as well. It is likewise important to cross-validate the measurement using new participants [87]. Further, some cultural differences could become apparent when testing the stability of the factor solution of the instrument [59]. Therefore, the MWBI needs to be tested in different cultures in order to verify its factor solution stability.

The small sample (8 participants) of the diary writing adds as a limitation for this study. The reason of the small sample of the diary writing is the eight participants were current bullying victims who have been identified after interviews. They were then invited to participant in diary writing to record their current bullying encounters. Although it is not seen as a major limitation because the recruitment of the sample was based on the objective of the study, it would seem unlikely that the full range of behaviors would be captured by such a small sample, or that sufficient exposure to such behaviors would occur with the small sample and short timeframe (one month). Further research studies are suggested to employ large sample and a large number of days per participant in order to make generalisation about bullying experiences across days and persons based on statistical power [88]. Therefore, it would be ideal to use this index in other samples by conducting broad-scale international comparative studies in other countries.

## Conclusions

The study reported here concluded that the 18-item MWBI is a reliable and valid means of measuring workplace bullying, and it contains two inter-related factors–work-related bullying and person-nature bullying. This mixed-method study entailed developing and validating a means for measuring bullying behavior in Eastern countries. The MWBI was produced using rigorous qualitative and quantitative approaches with reliable validation processes. The index is also suitable for multilevel data to access workplace bullying from a multilevel standpoint. Therefore, the MWBI deserves further studies for scale improvement and enrichment across countries to make the MBWI a universal measurement of workplace bullying.
